# Spontaneous Rupture of Leiomyoma Diagnosed Preoperatively with Noncontrast Computed Tomography

**DOI:** 10.1155/2020/5364165

**Published:** 2020-03-24

**Authors:** Angel Shan, Rahana Harjee, Patrick McLaughlin, Edward C. Jones, Mohamed A. Bedaiwy

**Affiliations:** ^1^Department of Obstetrics and Gynecology, Women's Hospital of British Columbia, University of British Columbia, Vancouver, Canada; ^2^Department of Radiology, Vancouver General Hospital, University of British Columbia, Vancouver, Canada; ^3^Department of Pathology, Vancouver General Hospital, University of British Columbia, Vancouver, Canada

## Abstract

**Background:**

Spontaneous rupture of benign uterine fibroids is extremely rare and has been associated with fibroid degeneration. It can cause acute intraperitoneal bleeding requiring immediate surgical intervention.

**Case:**

A previously healthy 50-year-old, Caucasian, nullipara presented with syncope, hemodynamic instability, and an acute abdomen. Noncontrast computed tomography images showed a positive sentinel clot sign in the pelvis as well as a large uterine fibroid with internal hyperdense clot suggesting acute rupture. Urgent laparotomy and hysterectomy confirmed a ruptured, actively bleeding, uterine fibroid with final pathological diagnosis of a benign leiomyoma.

**Conclusion:**

Prompt diagnosis and emergency surgical intervention were necessary to control acute hemorrhage from a ruptured uterine fibroid. Noncontrast computed tomography is an important adjunct to contrast-enhanced computed tomography and was vital for diagnosis in this case.

## 1. Introduction

Leiomyomas are common benign tumors and can undergo degeneration. Rupture of degenerated uterine leiomyomas to cause acute intraperitoneal hemorrhage is extremely rare with only case reports in the literature so far [1–10]. The pathological finding in these leiomyomas had significant degenerative changes or was associated with other factors such as infection [1], activity [2], and pregnancy [6, 7].

We present a case of a spontaneously ruptured benign uterine leiomyoma, without significant degenerative change, causing hemodynamic instability. Our case demonstrates the important role of noncontrast computed tomography (CT) and the imaging features of acute hemorrhage from a uterine fibroid that allowed for the prompt diagnosis of this rare but life-threatening event.

## 2. The Case

A 50-year-old nulliparous, premenopausal, previously healthy Caucasian woman presented to the emergency department with syncope, severe abdominal pain, and hypotension (blood pressure 80/40). Her symptoms began with rectal pressure. She bicycled to work as part of her normal morning routine. There was otherwise no history of trauma, intercourse, malignancy, abdominal surgeries, or known infection prior to presentation. Her past medical history included prior knee surgery and an umbilical hernia. She was not on any medications. There was a known history of a 7.5 cm fibroid incidentally found on a previous CT scan for evaluation of the umbilical hernia one year prior. Her menstrual cycles were regular, and she denied any history of abnormal PAP tests or sexually transmitted infections.

On examination, the patient had normal BMI. Her abdomen was peritonitic and actively distending over time with fluid resuscitation and packed red blood cell transfusion. Other than hemoglobin of 98 g/L, a complete blood count and comprehensive metabolic panel was within normal limits. The quantitative HCG was negative. A bedside ultrasound at the time of presentation by the emergency physician showed free fluid in her abdomen.

**Figure 1:**
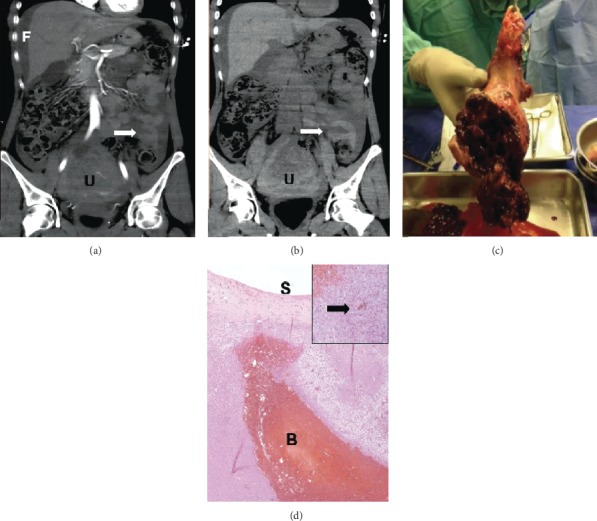
(a) Contrast-enhanced CT image with large volume hemoperitoneum (FF) and an enlarged uterus secondary to a large enhancing fibroid (UT). Sentinel blood clot in the pelvis with same enhancement as bowel loops nearby (arrow). (b) Noncontrast CT image of the retracted sentinel blood clot measuring 60 Hounsfield units (arrow). (c) Surgical specimen of the cervix and the uterus with a ruptured mass on the posterior aspect. (d) Highly cellular leiomyoma with blood (BL) tracking to the serosal surface (SS) and hemosiderin-laden macrophages (inset). Hematoxylin and eosin stain at high power magnification.

An emergency CT of the chest and abdomen was performed. Findings included moderate to large volume dense-free fluid (30 Hounsfield units) throughout the abdomen in keeping with large volume hemoperitoneum (Figure 1(a)). Noncontrast images showed a retracted sentinel clot in the pelvis measuring 60 Hounsfield units consistent with clotted blood, which was not distinguishable from normal enhancing myometrium and bowel wall on the contrast-enhanced images given the similar attenuation of enhanced muscle and clotted blood (Figure 1(b)). There was a significant interval enlargement of the uterus with the fibroid measuring 10.5 cm compared with 7.5 cm one year prior at the widest dimension. Within the myometrium and fibroid, there was a heterogeneous hyperdense attenuation with serpiginous hyperdense areas suggestive of clot and acute rupture (Figure 1(b)). Other intra-abdominal organs were unremarkable.

Exploratory laparotomy was undertaken and confirmed the imaging diagnosis. A large volume of blood and clots was found in the peritoneal cavity. An approximately 9 cm mass at the posterior aspect of the uterus was ruptured and actively bleeding (Figure 1(c)). The rest of the uterus, bilateral fallopian tubes, and ovaries appeared normal. The liver felt smooth, no adhesions or deposits were seen, and the bowel appeared normal on gross inspection. A total abdominal hysterectomy and bilateral salpingectomy was undertaken. A drain was placed intraoperatively and a cell-saver is used. The patient had an uneventful recovery and was discharged on postoperative day 3.

Pathological examination of the panhysterectomy specimen showed a 13 × 9 × 9 cm uterus with a benign uterine leiomyoma. The large mass seen on imaging was a 10.5 cm subserosal cellular uterine leiomyoma with fresh hemorrhage. On light microscopy, the leiomyoma was highly cellular but circumscribed without cytologic atypia, mitotic activity, or necrosis. It did not have significant degenerative changes. There was an intratumoral blood tracking to the uterine serosal surface. There was also evidence of previous subclinical bleeding with intratumoral hemosiderin-laden macrophages (Figure 1(d)). There were no underlying vascular abnormalities. The fallopian tubes and the cervix were unremarkable. There was an incidental well-differentiated endometrial adenocarcinoma, endometrioid type, grade 1/3, 1.7 cm in size, without myometrial invasion or involvement of the ruptured subserosal leiomyoma.

## 3. Discussion

Uterine leiomyomas are common benign tumors in women; however, complications resulting in acute hemoperitoneum are extremely rare. Large intraperitoneal hemorrhage from leiomyomas has been reported as a result of ruptured surface subserosal veins [8, 9]. Degeneration of leiomyomas resulting in rupture has also been described in both pregnant and nonpregnant patients [1–3]. In addition, cases of spontaneous perforation involving significant degeneration on pathologic finding occurred in the context of infection post dilation and curettage [1], ascites [10], and increased intra-abdominal pressure from physical activity [2]. Our case did not have degenerative changes of the leiomyoma and was not associated with any significant identifiable provoking factors.

The leiomyoma in this case was highly cellular on pathologic examination. It is possible that there was less stromal support and decreased tissue integrity. We speculate that there may have been a hemorrhage from small vessels within the fibroid that breached the tissue integrity and thus precipitated a rupture from within the mass. Whether this was immediately prior to the spontaneous rupture or a process that has been ongoing for a period of time with enlargement of the fibroid could not be determined.

Of note was the incidental finding of a small intramucosal endometrial adenocarcinoma in the uterus. It is unlikely to have contributed to the rupture as the leiomyoma was not involved. The leiomyoma had no pathologic findings of malignancy.

Various imaging modalities such as ultrasound, CT scan, and magnetic resonance imaging (MRI) have been used for evaluation of ruptured leiomyomas in previous case reports [2]. The acuity of presentation and instability of the patient limited the time available for imaging. In our case, CT scan alone was able to correctly identify the etiology of the large volume hemoperitoneum. The ability to distinguish blood clot, which has an inherent density of 50 HU-70 HU, from enhancing myometrium and collapsed bowel loops can be impossible after administration of iodinated contrast on a CT scan as enhancing muscle can have very similar attenuation. On the noncontrast images, a sentinel blood clot among bowel loops was identified in the pelvis suggesting a pelvic source of hemorrhage initially suspected to be of adnexal origin. A key finding on the noncontrast images was the presence of hyperdense linear areas of blood clot within the uterine fibroid and myometrium allowing preoperative diagnosis of a ruptured uterine fibroid with hemorrhage.

The lack of significant provoking factors, the acute onset large volume hemoperitoneum, and the lack of leiomyoma degeneration make our case unusual. In addition, we emphasize the importance of noncontrast CT scan images in promptly diagnosing the rupture and assisting surgical planning.

## 4. Conclusion

This case report describes that uterine fibroids can spontaneously rupture, without significant degeneration, to cause acute hemoperitoneum and require immediate explorative laparotomy and hysterectomy to control the hemorrhage. Noncontrast computed tomography is required to accurately visualize clotted blood, which is hyperdense, in the pelvis. Hyperdense collections of clotted blood, known as the “sentinel clot sign”, may be missed on contrast-enhanced studies due to its similar attenuation to enhancing myometrium and collapsed bowel wall on contrast-enhanced images.
